# Preconditioning with Hemin Decreases *Plasmodium chabaudi adami* Parasitemia and Inhibits Erythropoiesis in BALB/c Mice

**DOI:** 10.1371/journal.pone.0054744

**Published:** 2013-01-24

**Authors:** Esther Dalko, Véronique Gaudreault, Jaime Sanchez Dardon, Robert Moreau, Tatiana Scorza

**Affiliations:** 1 Basic and Clinical Immunology of Parasitic Diseases Group, Centre for Infection and Immunity of Lille and Institut National de la Santé et de la Recherche Médicale U547, Institut Pasteur de Lille, Lille, France; 2 Département des Sciences Biologiques, Université du Québec à Montréal, Montréal, Québec, Canada; Queensland Institute of Medical Research, Australia

## Abstract

Increased susceptibility to bacterial and viral infections and dysfunctional erythropoiesis are characteristic of malaria and other hemolytic hemoglobinopathies. High concentrations of free heme are common in these conditions but little is known about the effect of heme on adaptive immunity and erythropoiesis. Herein, we investigated the impact of heme (hemin) administration on immune parameters and steady state erythropoiesis in BALB/c mice, and on parasitemia and anemia during *Plasmodium chabaudi adami* infection. Intra-peritoneal injection of hemin (5 mg/Kg body weight) over three consecutive days decreased the numbers of splenic and bone marrow macrophages, IFN-γ responses to CD3 stimulation and T_h_1 differentiation. Our results show that the numbers of erythroid progenitors decreased in the bone marrow and spleen of mice treated with hemin, which correlated with reduced numbers of circulating reticulocytes, without affecting hemoglobin concentrations. Although blunted IFN-γ responses were measured in hemin-preconditioned mice, the mice developed lower parasitemia following *P.c.adami* infection. Importantly, anemia was exacerbated in hemin-preconditioned mice with malaria despite the reduced parasitemia. Altogether, our data indicate that free heme has dual effects on malaria pathology.

## Introduction

Malaria and other hemolytic disorders are characterized by enhanced susceptibility to bacterial and viral infections, and increased levels of IL-4, IL-6 and IL-10 [1], [2], [3]. These reports suggest a T helper (T_h_) 2 bias in chronic and acute hemolytic conditions that may contribute to altered cellular immunity. IFN-γ, a key cytokine for induction and maintenance of T_h_1 responses, is required for the clearance of intracellular bacteria and viruses, and for the elimination of *Plasmodium* parasites. In experimental murine malaria models, the sequential induction of an early T_h_1 response followed by a T_h_2 response is pivotal for the control of parasitemia and complete clearance of parasitized red blood cells (pRBCs) [4], [5]. The fact that concomitant infections with helminths enhance malaria morbidity by driving the differentiation of T_h_2 cells and the prevalence of the type 2 cytokines such as IL-4, and IL-13 [6], and that a potent IFN-γ response at the early phase of infection correlates with protection, suggest a protective role for T_h_1 responses in human malaria [4], [7]. CD4 T_h_1 cells are an important source of IFN-γ, a cytokine that activates monocytes and macrophages and promotes elimination of pRBCs by these phagocytes. Through IFN-γ, T_h_1 cells stimulate immunoglobulin class commutation and secretion of opsonizing antibodies by B cells, which reinforce parasite clearance by facilitating phagocytosis and antibody dependent cellular inhibition [8]. In contrast, exacerbated T_h_2 responses may be detrimental in malaria because IL-4, secreted by T_h_2 cells, suppresses T_h_1 differentiation and macrophage activation and drives the synthesis of non-opsonizing antibodies including IgE [9].

When hemolysis occurs, hemoglobin is released into the blood stream. During their development inside red blood cells (RBCs), *Plasmodium* parasites degrade up to 80% of the hemoglobin, the remaining of which is released during the rupture of pRBCs [10]. Free hemoglobin is rapidly oxidized to methemoglobin and releases its heme groups. It has been estimated that 10 healthy RBCs are eliminated for each pRBC, and that premature removal of RBCs is concurrent to the oxidation of membrane lipids caused by free heme, that render these cells fragile and senescent [11], [12], [13]. In addition, scavenger macrophages, or the deposition of autoimmune complexes mediated against RBCs are also involved in this process [13]. Increased methemoglobin levels are reported in human malaria, and murine *Plasmodium* infections also generate elevated levels of free heme [14], [15], [16], [17]. Various studies have reported mitogenic effects of heme on mouse and human CD4 T cells [18]. Heme also modulates the function of neutrophils, granulocytes, and macrophages [19], [20], and in this context, we reported that heme decreases the production of IL-12p70 by LPS–activated macrophages through an IL-10-dependent mechanism production, hampering also the stimulatory effects of IFN-γ on the IL-12p70 response [21].

Interestingly, various hemolytic conditions including malaria are characterized by dysfunctional erythropoiesis in the bone marrow. Severe malarial anemia (SMA) is of complex etiology and is concurrent to the lysis of pRBCs and non-parasitized RBCs, splenic sequestration of RBCs and bone marrow dysfunction [13], [22], [23]. SMA is often associated to reticulocytopenia, erythroid hyperplasia and increased levels of erythropoietin (EPO), suggesting a functional disruption of RBCs precursors [13], [23]. Impaired IL-12 responses and a systemic inflammatory response seem to be involved in this process [24], [25]. Accumulation of hemozoin in bone marrow macrophages has been linked to abnormal erythroid development, and patients with SMA have higher levels of hemozoin containing macrophages and lower levels of hemoglobin [13], [26]. In β-thalassemia, excess of α-globin chains and the associated heme induce reactive oxygen species (ROS) that contribute to the damage of mature and immature RBCs [27]. We have recently reported impaired growth of erythroid-burst forming unit (BFU-E) from bone marrow precursors from *Plasmodium chabaudi adami*–infected and phenylhydrazine-treated mice, which are accompanied by elevated ROS levels and high concentrations of circulating heme [28].

Considering that blunted T_h_1 immunity and dysfunctional erythropoiesis contribute to the pathogenesis of *Plasmodium* infection, and that heme seems to be a key modulator of these responses, we investigated the effects of pre-sensitization with hemin on CD4 T cells responses and erythroid parameters in naive BALB/c mice and in mice infected with *P. c. adami* DK parasites.

## Materials and Methods

### Mice, Treatment and Infection

Four- to 6-weeks old female BALB/c mice (Charles River Laboratories, Montreal, QC, Canada) were used in all experiments. To evaluate the effect of hemin *in vivo*, mice were injected intraperitoneally with 5 mg/Kg hemin for three days and control mice were treated with PBS. Hemin (porcine; Sigma Aldrich, Canada) was prepared by dissolving a stock solution of 25 mg/mL hemin (in 0.1 M NaOH) in PBS. Mice were euthanized 24 h after the last injection. Naive and hemin-treated mice were infected with 10^5^ *Plasmodium chabaudi adami* DK-parasitized RBCs by the intravenous route. Parasitemia was estimated daily from tail blood smears strained with 10% Giemsa solution. All the manipulations in mice were conducted according to relevant national and international guidelines. All procedures in mice were approved by the Animal Care Committee of the Université du Québec à Montréal (protocol 0511-720-0512).

### Assessment of Cell Populations in the Spleen, and Bone Marrow

Cells were recovered from the spleen and the bone marrow and stained with anti-TER119 and anti-CD71 antibodies (BioLegend) to evaluated erythroid populations. After lysing RBCs with the RBC lysing buffer Hybri-Max® (Sigma Aldrich, Canada), single-cell suspensions of splenocytes were treated with anti CD4-FITC and anti-F4/80-PE antibody (BioLegend) to respectively assess the number of CD4 T cells and macrophages. Cells were further kept for 30 min at 4°C and fluorescence was analyzed on a FACScan flow cytometer (Becton Dickinson, USA). Data provided by 10,000 events were analyzed using the FlowJo software.

### Cell Culture and Purification

Spleen cells from naive and hemin-treated mice were recovered, RBCs were lysed with the RBC lysing buffer Hybri-Max®, and splenocytes were cultured in complete culture medium (RPMI 1640 supplemented with 10% FBS, 10 U/mL streptomycin and penicillin and 5×10^−5^ M β-mercaptoethanol) at 4×10^6^ splenocytes/mL. CD4 T cells were purified using the EasySep mouse CD4 T cell Enrichment Kit (StemCell Technologies, Canada). Purity levels were ≥85% as assessed by flow cytometry.

### Quantification of Activated CD4 T Cells and Secretion of IFN-γ/IL-4

To assess the cytokine response, 4×10^6^ splenocytes/mL spleen cells were cultured in 48-well plates coated overnight with anti-CD3 (2.5 µg/mL) monoclonal antibody (eBioscience). After 48 h of stimulation, IFN-γ and IL-4 concentrations were measured in culture supernatants with specific ELISA MAX™ Deluxe Sets (BioLegend). The same protocol was used with 2×10^6^ purified CD4 T cells/mL stimulated with 2.5 µg/mL coated anti-CD3 and 1.5 µg/mL soluble anti-CD28 monoclonal antibodies (eBioscience).

### *In Vitro* T_h_ Cell Differentiation

Purified CD4 T cells from naive and hemin-treated mice were cultured at 0.5×10^6^ cells/mL in 24-well plates pre-coated with anti-CD3 (0.2 µg/mL) and soluble anti-CD28 (1.5 µg/mL) monoclonal antibodies. For T_h_1 differentiation, CD4 T cells were stimulated in the presence of recombinant IL-12 (30 ng/mL; BioLegend) and anti-IL4 antibody (10 ng/mL; BioLegend). For T_h_2 differentiation, the cells were stimulated in the presence of recombinant IL-4 (40 ng/mL; BioLegend), anti-IL-12 (10 µg/mL; BioLegend) and anti-IFN-γ (10 µg/mL; BioLegend) antibodies. After three days of stimulation, recombinant IL-2 (10 ng/mL; BioLegend) was added to the medium and cells were cultured for 3 additional days. The cells were then recovered, washed once in RPMI 1640 and further incubated with anti-CD3 monoclonal antibody (0.3 µg/mL) for 24 h, after which IFN-γ and IL-4 levels were assessed by ELISA (ELISA MAX™ Deluxe Sets; BioLegend) in the supernatant.

### Determination of Haemoglobin and Reticulocyte Levels in Plasma

Hemoglobin concentrations were measured by diluting 2 µl tail-vein blood in 500 µL Drabkin’s solution. Two hundred microliters were split in 96-well plates in duplicate and the absorbance was measured at 540 nm with a microplate reader. Values were converted to g/dL using a standard curve of pure hemoglobin dissolved in Drabkin’s solution. In order to assess the percentage of reticulocytes in the blood, 2 µl of blood from tail-tip were collected in 500 µL of PBS. The blood cell suspensions were centrifuged and resuspended in 250 µL of a 0.025% glutaraldehyde solution overnight. The cell suspensions were then stained with anti-CD71-FITC antibody (BioLegend), incubated at 4°C for 30 min and fluorescence was measured on a FACScan flow cytometer (Becton Dickinson, USA). Data were analyzed using the FlowJo software.

### Determination of Heme, Hemopexin and Erythropoietin Concentrations in Plasma

Plasma was recovered from euthanized mice in a heparinized eppendorf. Total heme levels were measured in the plasma using its elicited luminescence. Briefly, 5 µL of plasma was diluted in 25 µL PBS and was added to an opaque 96-well plate. A luminescence reader (MLX microtiter plate luminometer with the software Revelation MLX, Version 4.27, Dynex Technologies, Chantilly, VA, USA) was set to dispense simultaneously 100 µL of a 1 µg/mL luminol solution (dissolved in 0.1 M NaOH and 3 mM EDTA; Sigma Aldrich, Canada) and 100 µL of a 7.26 mM t-buthylhydroperoxide solution (dissolved in 0.1 M NaOH and 3 mM EDTA; Sigma Aldrich, Canada). Luminescence output was measured immediately with a 2 s interval. A standard curve was made with the stock solution of heme to express results in µM. To assess hemopexin concentrations in the plasma the Mouse Hemopexin ELISA kit (Kamiya Biomedical Company) was used; the Mouse EPO ELISA kit (Kamiya Biomedical Company) was used for EPO level measurement in the plasma.

### Colony-forming Unit Assays

Single-cell suspensions were prepared from the spleen and bone marrow and RBCs from the spleen were lysed in RBC Lysing buffer Hybri-Max®. Two million cells/mL were prepared in RPMI 1640 and 0.3 mL were added to 3 mL of MethoCult® 03334 medium for duplicate cultures, as described by the manufacturer. Cultures were incubated at 37°C, 5% CO2 in air and ≥95% humidity for 8 days for optimal quantification of erythroid-blast forming units (BFU-E) (Figure S1).

### Statistical Analysis

Statistical analyses were performed using a nonparametric Student *t* test for comparison of activation and cytokine responses between naive and hemin-treated cells using the Prism software; *p*<0.05 was considered as significant.

## Results

### Impact of Hemin Administration on Spleen Cells and Cytokine Responses

**Figure 1:**
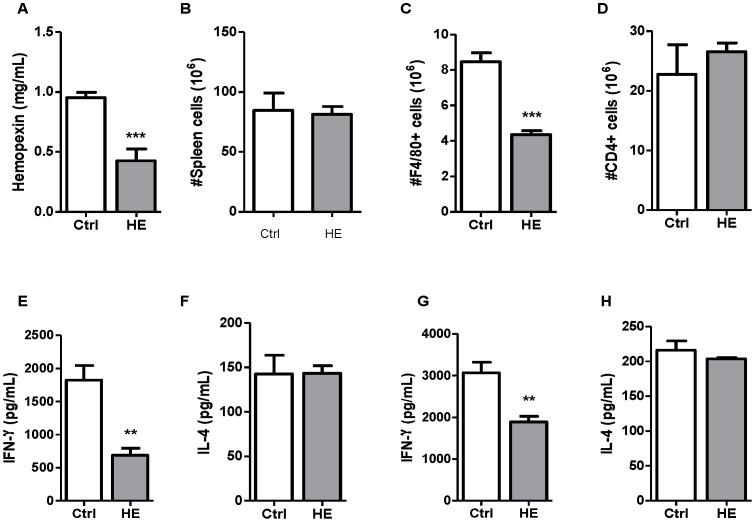
Hemin-mediated immunomodulation. Hemin (HE; 5 mg/kg) was injected consecutively during three days whereas control (Ctrl) mice received PBS. Hemopexin levels were assessed in the plasma by ELISA (A). Spleens were recovered and total numbers of cells (B) were determined. The numbers of macrophages (F4/80^+^ cells) (C) and CD4 T cells (D) were estimated following staining with specific antibodies and analysis by flow cytometry. Total spleen cells from control and HE-treated mice were stimulated with anti-CD3 monoclonal antibody for 48 h to assess production of IFN-γ (E) and IL-4 (F) by ELISA. In parallel, purified CD4 T cells were cultured in T_h_1 (G) and T_h_2 (H) polarizing conditions as described in the *Material and Methods* section. Data in A and G are mean ± SEM from one experiment (n = 3–9 mice per group); results from B to F are mean ± SEM from two independent experiments (n = 6–10). Values were compared using a non-parametric Student *t* test. **p<0.01; ***p<0.001.

High concentrations of free heme are characteristic of hemolytic disorders, which are pathologies associated with altered immunity. Considering the importance of activated macrophages and IFN-γ for the control of blood stage malaria, the *in vivo* effects of hemin on mouse macrophages and CD4 T cells were investigated. Hemopexin is the most important scavenger of free heme, and its levels decrease in hemolytic pathologies including malaria [29], [30]. At a dose of 5 mg/Kg body weight, three consecutive injections of hemin decreased the plasmatic levels of hemopexin in mice, confirming partial saturation of this scavenging system (Fig. 1A). Total numbers of spleen cells were comparable in control and hemin-treated mice (Fig. 1B).The numbers of macrophages (F4/80^+^ cells) significantly dropped in the spleen of mice injected with hemin compared to control mice (Fig. 1C), whereas the numbers of splenic CD4 T cells were not affected (Fig. 1D).

Splenocytes from naive and hemin-treated mice were stimulated *ex-vivo* with anti-CD3 antibody in order to investigate the effects of hemin conditioning on T cells responses. Total spleen cells from hemin-treated mice produced significantly less IFN-γ compared to naive cells (Fig. 1E), whereas IL-4 levels remained comparable in hemin-treated and control mice (Fig. 1F). A similar trend was observed in purified CD4 T cells cultured under T_h_1 and T_h_2 polarizing conditions, as the production of IFN-γ was significantly decreased in T_h_1 cells from hemin-treated mice (Fig. 1G) but the IL-4 response was not affected in T_h_2 polarized CD4 T cells (Fig. 1H).

### Bone Marrow and Spleen Erythropoiesis

**Figure 2:**
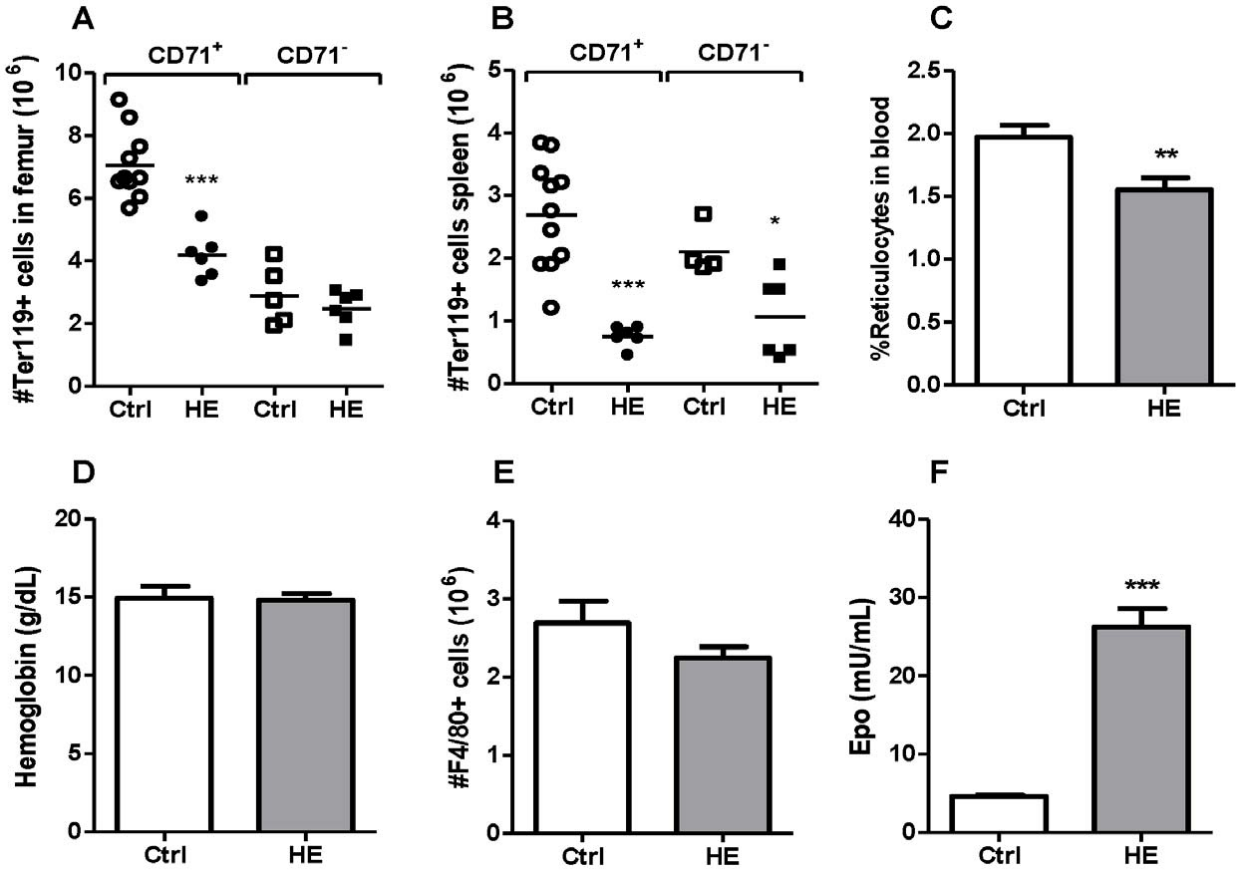
Bone marrow and spleen erythroid parameters. Control (Ctrl) and hemin (HE)-treated mice were euthanized 24 h after the last injection with PBS or HE. Populations of erythroid cells were analyzed in the bone marrow (A) and in the spleen (B) by staining cells with anti-CD71-FITC and anti-Ter119-PE antibodies. The percentage of reticulocytes (CD71^+^ cells) (C) and hemoglobin levels were measured in the blood (D). Macrophages (F4-80^+^ cells) were quantified in femoral bone marrow cell suspensions (E) by staining with anti-F4/80-PE antibody. Erythropoietin (EPO) was measured in the plasma by ELISA (F). Data are mean ± SEM from two independent experiments (n = 4–11 mice per group) and values were compared using a non-parametric Student *t* test. *p<0.05; **p<0.01; ***P<0.001.

In addition to immune parameters, the impact of hemin on erythroid cells was investigated by staining cells with anti-CD71-FITC and anti-TER119-PE antibodies. Decreased numbers of erythroid cells expressing the transferring receptor (CD71^+^Ter119^+^) were measured in the bone marrow of hemin-conditioned mice, whereas the numbers of mature erythroid cells (CD71^-^Ter119^+^ cells) were not affected (Fig. 2A). Fewer erythroid cells were found in the spleen of hemin-treated mice in respect to the control group (Fig. 2B), suggesting effects in splenic erythropoietic niches in steady state conditions. The percentages of circulating reticulocytes were significantly reduced in mice treated with hemin although hemoglobin levels were not affected (Fig. 2C and 2D).

Considering that macrophages play important roles in erythropoiesis [31], we evaluated the impact of hemin on the population of resident macrophages (F4/80^+^ cells) in the bone marrow, and no significant effect was revealed with the dose of hemin tested (Fig. 2E). Interestingly, the levels of EPO in plasma increased more than 3-fold in hemin-treated mice compared to naive mice (Fig. 2F).

### Impact of Hemin Treatment on Plasmodium Adami Infection

**Figure 3:**
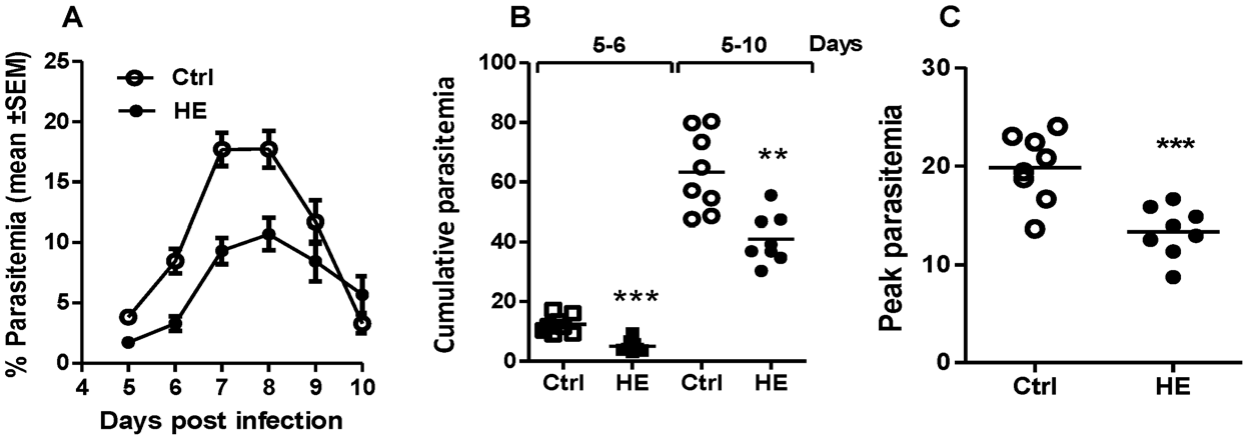
Impact of heme on the clearance of *Plasmodium* infection. *P. chabaudi adami* DK (10^5^ parasitized RBCs) were inoculated by the intravenous route in PBS (Ctrl)- and hemin (HE)-preconditioned mice (5 mg/kg, for 3 consecutive days) 24 h after the last injection. Parasitemia was followed daily from tail-tip blood smears for estimation of the kinetics of infection (A) and cumulative (B) and peak parasitemia (C). Values represent the mean ± SEM from two independent experiments (n = 8) and were compared using a non-parametric Student *t* test. **p<0.01; ***p<0.001.

Based on the decreased ability of CD4 T cells to secrete IFN-γ and to differentiate into T_h_1 cells and the reduced numbers of splenic macrophages found in hemin-conditioned mice, we hypothesized that administration of hemin would exacerbate *P. c. adami* infection. Following injection of PBS or hemin over three days, mice were infected with *P. c. adami* DK parasites and parasitemia was followed daily. Unexpectedly, a significant decrease in parasitemia was measured in hemin-treated mice in two independent experiments, which are represented as compiled results in figure 3A. Cumulative parasitemia, estimated as the sum of daily parasitemia throughout the period of patent infection, was significantly lower at early infection (days 5–6) and throughout patent infection (days 5–10) in hemin-treated mice (Fig. 3B), and peak parasitemia was also significantly decreased (Fig. 3C).

### Splenic Response and Erythroid Precursors in Hemin-conditioned Mice with Malaria

**Figure 4:**
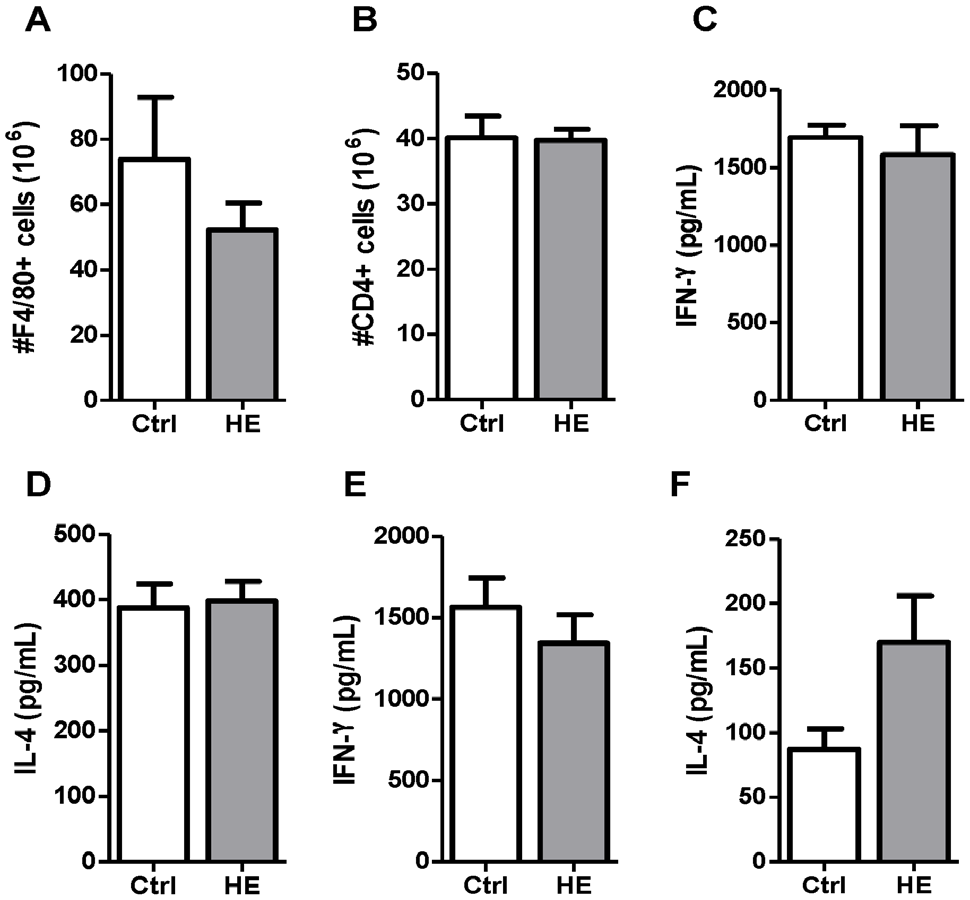
Immune parameters in control and heme-preconditioned BALB/c mice 10 days after *P. c. adami* infection. PBS (Ctrl) and hemin (HE)-treated mice were euthanized and spleen was recovered to assess the number of macrophages (F4/80^+^ cells) (A) and CD4 T cells (B). Spleen cells were stimulated with anti-CD3 monoclonal antibody for 48 h to assess IFN-γ (C) and IL-4 (D) production by ELISA. Purified CD4 T cells were stimulated with anti-CD3 and anti-CD28 monoclonal antibodies to estimate the production of IFN-γ (E) and IL-4 (F). Data represent the mean ± SEM from two independent experiments (n = 7–12) and values were compared using a non-parametric Student *t* test.

Considering the blunted IFN-γ responses measured in hemin-conditioned mice, a comparable analysis of the populations and cytokine responses was done with splenic cells from control and hemin-treated mice at day 10 post-infection. At this time point, parasitemia is significantly decreased and erythropoiesis is readily enhanced. Macrophage numbers were comparable in control and hemin-treated mice at day 10 post-infection (Fig. 4A) and no significant differences were measured in respect to CD4 T cell numbers (Fig. 4B) or cytokine responses of total splenic (Fig. 4C, D) and purified CD4 T cells (Fig. 4 E, F).

**Figure 5:**
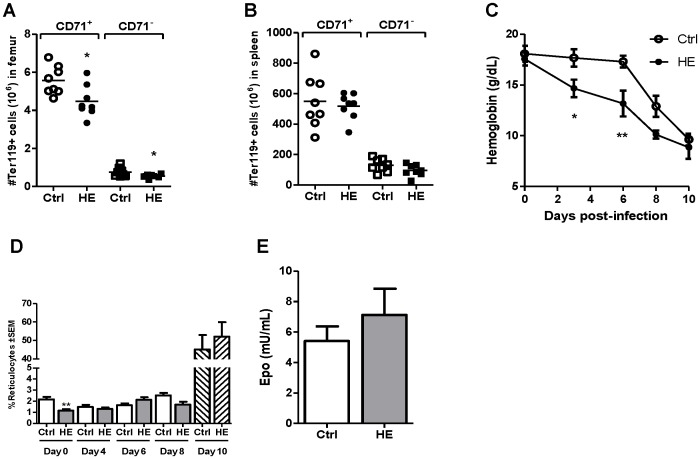
Bone marrow and spleen erythroid parameters in control and heme pre-conditioned mice infected with *P. c. adami*. Control (Ctrl) and heme (HE)– preconditioned mice were infected with *P. chabaudi adami* (10^5^ parasitized RBCs) and euthanized at day 10 post-infection. The number of erythroid precursors was estimated in the bone marrow (A) and spleen (B) with anti-CD71-FITC and anti-Ter119-PE antibodies. Hemoglobin levels (C) and percentages of reticulocytes (D) were determined in blood daily, and plasmatic concentrations of EPO were quantified at day 10 post infection (E). Values in A-D are the mean ± SEM from two independent experiments (n = 8). Data from (E) are the mean ± SEM from one experiment (n = 4). Values were compared using a non-parametric Student *t* test. *p<0.05; **p<0.01.

Despite the drop in parasitemia measured in hemin-conditioned mice, significant lower numbers of bone marrow CD71^+^ and CD71^+^ erythroid cells were found in these mice (Fig. 5A). In contrast, no major differences were observed in the splenic erythroid populations (Fig. 5B)). Although comparable levels of hemoglobin were measured at day 10 post-infection in control and hemin-conditioned mice, hemoglobin levels were lower in hemin-preconditioned mice at day 6 post-infection when compared to controls (Fig. 5C). The percentages of circulating reticulocytes were low in hemin-conditioned mice the day of infection, but thereafter became comparable to controls, reaching their highest values at day 10 post-infection (Fig. 5D). These results show that despite developing lower parasitemia, anemia is relatively exacerbated in mice pre-conditioned with hemin at early infection. Comparable EPO levels were found in the plasma of control and hemin-preconditioned mice at day 10 post-infection (Fig. 5E).

## Discussion

The inflammatory response and systemic oxidative stress generated in hemolytic pathologies is accompanied by important alterations in immunity and dysfunctional erythropoiesis. The levels of the T_h_2 type cytokines IL-4, IL-10 and IL-13 are relatively enhanced whereas IFN-γ responses are compromised in thalassemia, sickle cell disease (SCD) and autoimmune hemolytic anemia [1], [2], [3]. Malaria is a hemolytic disease also characterized by high levels of free heme in circulation, oxidative stress and inflammation [16], [32], and *Plasmodium*-infected hosts are particularly susceptible to bacterial and viral infections, as are people with genetic hemolytic disorders [33], [34], [35], [36]. Impaired IFN-γ responses and high IgE levels are found in people with severe malaria [37], [38], whereas mild malaria correlates with higher IFN-γ and high levels of opsonizing antibodies [39], [40], indicating that a predominant T_h_2 bias is detrimental and that T_h_1 immune responses are protective. In disorders characterized by chronic hemolysis, free hemoglobin from damaged RBC is rapidly oxidized into methemoglobin and releases its heme groups [16]. In circulation, free heme binds easily to cell membranes due to its lipophilic nature and is also rapidly complexed to serum albumin [41]. Accumulation of heme is concurrent to overwhelmed hemopexin scavenging, the main mechanism sequestrating heme and recycling its iron [29]. Herein, hemin was injected in mice at concentrations that reduced 50% the plasmatic levels of hemopexin, indicating saturation of this scavenger, as found in patients with SMA [30] and other hemolytic conditions [29]. Indeed, low levels of hemopexin are found in SCD and thalassemia [29], whereas levels of heme are high in the plasma of *Plasmodium*-infected mice [15], [42]. Administration of hemin dampened IFN-γ responses and decreased the numbers of splenic macrophages. However, lower parasitemia was measured in hemin-preconditioned mice following infection with *P. c. adami* DK parasites, which was accompanied by exacerbated anemia at early infection. Despite the impaired IFN-γ responses and dyserythropoiesis concurrent to hemin pre-treatment, administration of hemin seems detrimental for the development of blood stage parasites.

Various factors can contribute to the altered IFN-γ responses found in hemin-conditioned mice. The redox status of cells involved in innate and adaptive immune responses may significantly influence cytokine responses in macrophages and T cells. Macrophages treated with hemin secrete less IL-12p70 and more IL-10, which are effects associated with high intracellular levels of ROS and decreased concentrations of reduced glutathione [21], [43]. In this context, it has been reported that the reduced/oxidized glutathione ratio of antigen presenting cells influences the polarization of CD4 T cells into T_h_1 or T_h_2 subsets [44]. Accordingly, our data reveal that administration of hemin dampens the production of IFN-γ by spleen cells as well as by CD4 T cells cultured under stringent conditions for T_h_1-polarization.

Early secretion of IFN-γ by CD4 T cells is of particular importance for clearance of *P*. *chabaudi adami* DK parasites [4], [5], [7]. Although having compromised production of IFN-γ prior to infection, hemin-conditioned mice developed milder infection than control mice. These results are in agreement with the protective effect of hemolytic disorders on high rates of parasitemia. Indeed, SCD and glucose-6-phosphate deficiency are traits associated with low malaria parasitemia, but the mechanisms involved are not yet fully understood. Considering that the inhibitory effects on parasitemia measured in hemin-preconditioned mice were apparent at early infection, it seems plausible that hemin renders RBCs less prompt to infection by merozoites or less apt in generating high numbers of merozoites during the first waves of parasite replication. By affecting the rigidity of RBC membranes heme may interfere with parasite penetration in the RBC [45], since it intercalates in the membrane bi-layer and oxidizes membrane phospholipids. The negative impact for hemin on *P. c. adami* DK parasitemia greatly contrasts with the absence of effect of hemin reported by Ferreira et al. on *P. berghei* parasitemia [42]. Unlike *P. c. adami*, *P. berghei* invades reticulocytes and mature RBCs, and it should be expected that the lower proportions of reticulocytes measured in hemin-preconditioned mice are detrimental for *P. berghei* development [46]. However, reticulocytes are more equipped to cope with oxidative damage than mature RBCs, as they possess higher concentrations of anti-oxidants [47], and hemin may affect more drastically mature RBCs. In this context, our analysis revealed lower numbers of macrophages in hemin-treated mice, an effect that may be concurrent to enhanced elimination of prematurely senescent/damaged RBCs and concomitant apoptosis of macrophages, or to direct toxic effects of hemin on macrophages, as described for RBCs and astrocytes [48], [49]. Indeed, hemin promotes exposure of phosphatidylserine in the outer surface of the RBC [48], which favors clearance of RBCs by macrophages and which in turn may contributes to their apoptosis [50].

In mice, homeostatic erythropoiesis occurs in the bone marrow, whereas stress erythropoiesis is primarily assured by the spleen [13]. During *P. c. adami* infection, splenic erythropoiesis resulted in more than 200 fold increase in immature erythroid progenitors in the spleen, both in control and hemin-treated mice, and although the percentages of reticulocytes were lower in hemin-preconditioned mice prior to infection, reticulocytemia was efficiently restored. As significantly lower numbers of immature and mature erythroid cells were found in the bone marrow of hemin-preconditioned mice prior to and 10 days after infection, we speculate that the relatively exacerbated anemia measured in hemin-conditioned mice at early infection is concurrent to an impaired ability of the bone marrow in responding to erythropoietic stress. We speculate that as anemia accentuates throughout infection, the spleen becomes engaged in generating new RBCs to compensate for the limited capacity of bone marrow erythropoietic niches to expand.

The importance of macrophages in erythropoiesis has been underscored by the fact that treatment with liposome-encapsulated dichloromethylene biphosphonate depletes femoral macrophages and results in inefficient generation of blast forming erythroid units (BFU-E) *ex-vivo* [31]. Although administration of hemin during 3 consecutive days failed in altering the numbers of bone marrow macrophages, treatment with hemin for an additional week decreased significantly the numbers of macrophages in the femoral bone marrow (Figure S1). Prolonged administration of hemin also decreased hemoglobin levels and impaired the generation of bone marrow derived BFU-E *ex-vivo* (Figure S1). We suggest that the hemolysis induced by *P. c. adami* infection in conjunction with pre-conditioning with hemin exacerbated the detrimental effects of this oxidant on bone marrow macrophages and erythropoiesis.

The rise in EPO levels measured in mice treated with hemin for 3 consecutive days suggests alterations in EPO production by the kidney, which is normally stimulated by hypoxia [51]. Interestingly, renal and extra-renal production of EPO is enhanced in mice suffering from hemolytic anemia when compared to bled mice, suggesting that factors released by RBCs may influence EPO responses [52]. Accordingly, enhanced erythropoiesis has been reported in dogs and rats injected with hemolysates [53]. Heme induces the expression of heme oxygenase-1, which in turn, catalyzes its degradation into carbon monoxide and biliverdin. As carbon monoxide has a higher affinity with hemoglobin than oxygen, it decreases the capacity of RBCs to carry O_2_, and it is also possible that carbon monoxide resulting from heme degradation through heme oxygenase-1 may contribute to hypoxia, which in turn stimulates renal production of EPO [54].

In conclusion, our data indicate that hemin inhibits the production of IFN-γ by spleen cells, and induces bone marrow dyserythropoiesis, which in mice may be efficiently compensated by the spleen. As preconditioning with hemin reduced parasitemia, our model also suggests that clearance of blood-stage *Plasmodium* infections is favored by hemolysis and that free hemin may have both detrimental and beneficial effects in blood stage malaria.

## Supporting Information

Figure S1 **Mice were injected with PBS (Ctrl) or hemin (HE; 5 mg/kg) 3 times a week for 2 weeks.** Hemoglobin concentrations in blood (A) and hemopexin concentrations in plasma (B) were determined 24 h after the last injection. Cells from femoral bone marrow (BM) and splenocytes (SPL) were recovered and stained with anti-F4/80-PE antibody to estimate the number of macrophages (C), and reticulocytes were determined in blood cells with anti-CD71-FITC antibody (D). BFU-E cells were quantified in BM (E) and spleen cell cultures (F), as described in the *Material and Methods section*. Data represent 6–13 mice per group, and were compared using a non-parametric Student *t* test. *p<0.05, **p<0.01; ***p<0.001.
(TIFF)
