# Supplementary figures and images for: Preconditioning with Hemin Decreases Plasmodium chabaudi adami Parasitemia and Inhibits Erythropoiesis in BALB/c Mice
Source: PLoS One. 2013 Jan 24;8(1):e54744. doi: 10.1371/journal.pone.0054744 (PMC3554635; doi:10.1371/journal.pone.0054744)

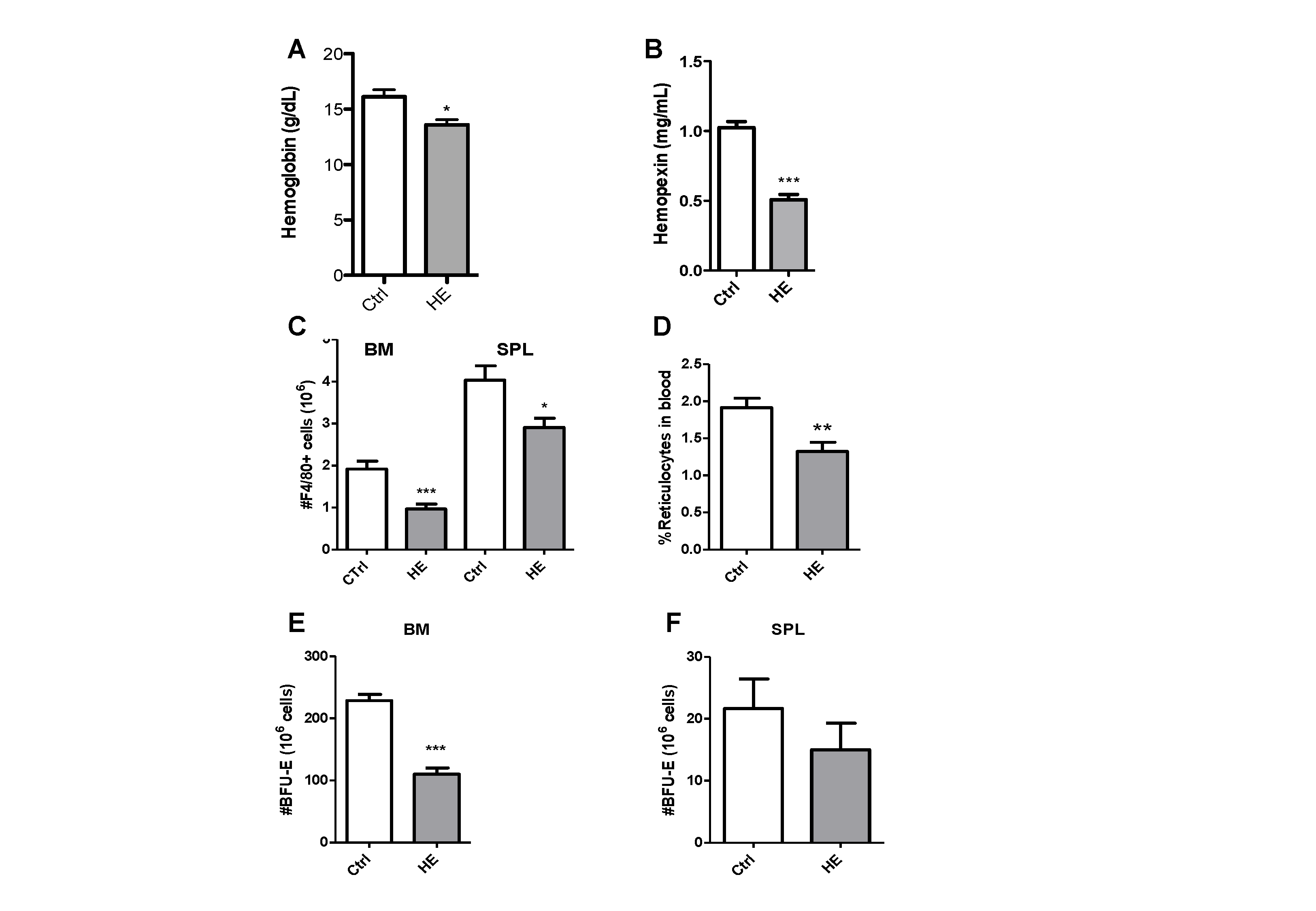

Supplement: Figure S1 — Mice were injected with PBS (Ctrl) or hemin (HE; 5 mg/kg) 3 times a week for 2 weeks. Hemoglobin concentrations in blood (A) and hemopexin concentrations in plasma (B) were determined 24 h after the last injection. Cells from femoral bone marrow (BM) and splenocytes (SPL) were recovered and stained with anti-F4/80-PE antibody to estimate the number of macrophages (C), and reticulocytes were determined in blood cells with anti-CD71-FITC antibody (D). BFU-E cells were quantified in BM (E) and spleen cell cultures (F), as described in the Material and Methods section. Data represent 6–13 mice per group, and were compared using a non-parametric Student t test. *p<0.05, **p<0.01; ***p<0.001. (TIFF) [file pone.0054744.s001.tiff]
